# Assessment of the Impact of the Recombinant Porcine Reproductive and Respiratory Syndrome Virus Horsens Strain on the Reproductive Performance in Pregnant Sows

**DOI:** 10.3390/pathogens9090772

**Published:** 2020-09-21

**Authors:** Sandra Genís, Lise K. Kvisgaard, Lars E. Larsen, Lucas P. Taylor, Jay G. Calvert, Mònica Balasch

**Affiliations:** 1 Zoetis Manufacturing & Research Spain S.L., Ctra Camprodon s/n, 17813 Finca La Riba, Spain; sandra.genis@zoetis.com; 2 National Veterinary Institute, Technical University of Denmark, 2800 Lyngby, Denmark; likik@sund.ku.dk (L.K.K.); lael@sund.ku.dk (L.E.L.); 3 Department of Health and Medical Sciences, Institute for Veterinary and Animal Sciences, University of Copenhagen, 1870 Frederiksberg, Denmark; 4 Zoetis Inc., 333 Portage St, Kalamazoo, MI 49007, USA; Lucas.p.taylor@zoetis.com (L.P.T.); jay.calvert@zoetis.com (J.G.C.)

**Keywords:** PRRS, MLV, recombinant

## Abstract

This study assessed the impact of a PRRSV (porcine reproductive and respiratory syndrome virus) recombinant strain (Horsens strain) on the reproductive performance of naïve pregnant sows in the last third of gestation. Fifteen sows were included: four negative reproductive controls (NTX), five infected with a PRRSV-1 field strain (Olot/91, T01), and six infected with the recombinant PRRSV-1 strain (Horsens strain, T02). Piglets were monitored until weaning. Reproductive performance was the primary variable. In sows, viremia and nasal shedding (T01 and T02 groups), and, in piglets, viral load in blood and in lungs, as well as macroscopic lung lesions (T01 and T02 groups), were the secondary variables. The reproductive performance results were numerically different between the two challenged groups. Moreover, viral loads in blood were 1.83 × 10^6^ ± 9.05 × 10^6^ copies/mL at farrowing, 1.05 × 10^7^ ± 2.21 × 10^7^ copies/mL at weaning from piglets born from T01 animals and 1.64 × 10^3^ ± 7.62 × 10^3^ copies/mL at farrowing, 1.95 × 10^3^ ± 1.17 × 10^4^ copies/mL at weaning from piglets born from T02 sows. Overall, 68.8% of T01 piglets and 38.1% of T02 piglets presented mild lung lesions. In conclusion, the results suggest that Horsens strain is less virulent than the field strain Olot/91 under these experimental conditions.

## 1. Introduction

Porcine reproductive and respiratory syndrome virus (PRRSV) is a highly infectious small, enveloped, positive-sense single-stranded RNA virus, which is endemic in almost all pork producing areas of the world. The first described outbreaks of PRRS in Europe were in 1990 in Germany. The etiological agent, PRRSV, was identified in Europe in 1991 (prototype: “Lelystad virus”) [1]. Later, a related but genetically divergent PRRSV was isolated in the USA (prototype: “VR-2332”) [2]. PRRSV is currently classified as PRRSV-1 (formerly European genotype 1) and PRRSV-2 (formerly North American genotype 2) species. The genomic nucleotide sequences of PRRSV-1 and PRRSV-2 differ by approximately 44% [3].

Since 1991, PRRSV-1 has spread throughout Europe and into North America and Asia, while steadily increasing in genetic diversity. The severity of PRRS disease can vary widely from subclinical to devastating outbreaks of reproductive diseases in sows (anorexia, fever, lethargy, nervous signs, abortion, stillbirths) and lethal respiratory disease in young pigs [1].

Currently, PRRS is considered to be among the diseases with the highest economic impact in the modern pig industry [4]. In Europe and North America, the cost of PRRSV to the industry has been estimated at $6.25–$15.25 USD per pig marketed [4,5].

There are currently at least 18 commercially licensed modified live vaccines (MLVs) for the control of PRRS in the global market, including at least six in Europe. Because these vaccines are live viruses that retain their ability to spread from pig to pig and to undergo genetic changes, including recombination, precautions need to be followed in order to maintain their safety. None of these MLVs are labeled for use in PRRSV-negative or naïve herds due to residual reproductive virulence in naïve sows late in pregnancy.

Homologous recombination is the process by which related segments of genetic material (RNA or DNA) may be exchanged between related organisms. This process occurs naturally in essentially all microorganisms and is believed to be important for the evolution of species [6]. Recombination enables different beneficial mutations in separate genomes to be combined into a single genome, resulting in an organism that shows advantages over its predecessors in terms of replication potential and survival (i.e., increased “fitness”). Evidence that homologous recombination occurs at high frequency in PRRS virus genomes was reported more than two decades ago [7,8]. Therefore, the simultaneous or contemporaneous use of different PRRSV MLV vaccines poses a risk of recombination. Renson et al. reported a recombinant PRRSV-1 strain derived from two MLV vaccines (Unistrain PRRS and Porcilis PRRS) in a pig farm in France [9]. In a later study from the same group, animals infected with the recombinant strain did not show significant clinical signs, although the recombinant strain demonstrated increased excretion and transmission capabilities compared to parental vaccine strains [10]. Other recombinant strains that have been identified to date have derived from the recombination between PRRSV field strains [11,12] or between field strains and MLV vaccine strains [13,14,15].

In July 2019, PRRSV-1 was detected in semen samples taken as part of the routine PRRSV surveillance in a PRRSV-negative boar station in Denmark [16]. More than 650 production herds, including many PRRS-negative herds, received semen from the infected boar station. It was reported that the virus seemed to be highly transmissible and caused severe disease in infected herds, with clinical signs similar to, or even exceeding, those normally observed in Danish herds infected with PRRSV-1 [16]. Kvisgaard et al. reported that the clinical signs included severe reproductive problems and abortions, significant increases in post-farrowing mortality, and respiratory disease in young pigs. Furthermore, the viral load found in processing fluids, lung, and serum of infected pigs exceeded the levels normally observed in samples from pigs with PRRSV, indicating a high level of viral replication. The same research group reported that this new PRRSV-1 strain was derived from homologous recombination between two PRRSV-1 MLV strains (Suvaxyn PRRS MLV, Zoetis, Genbank accession MK876228, and Unistrain PRRS, Hipra Genbank accession GU067771).

The objective of this study was to assess the impact of the new PRRSV recombinant strain (Horsens strain, Genbank accession MN603982) on reproductive performance in seronegative sows infected in the last third of gestation in comparison with a field PRRSV-1 isolate of proven reproductive pathogenicity [17]. The final objective was to determine if the described increased virulence was verified under experimental conditions.

## 2. Materials and Methods

### 2.1. Ethics Statement

This experiment was conducted in the BSL2 facilities of Zoetis Manufacturing & Research Spain S.L. in Olot, Spain. All experimental procedures were approved by Zoetis Animal Welfare Committee. 

### 2.2. Virus Strains

PRRSV Olot/91 (ECACC accession number V93070108, Genbank accession KF203132) was isolated from a stillborn piglet born from a PRRSV-infected sow in 1991 [17] and propagated in PAM culture (pulmonary alveolar macrophages). The vial used corresponded to a passage 4 with a titer of 10^4^ TCID_50_/mL.

Horsens strain was provided by the Technical University of Denmark, Lyngby, Denmark. The strain was isolated from serum samples from infected animals [16] and propagated in MARC-145 cells. The vial used corresponded to passage 3 with a titer of 10^8^ TCID_50_/mL.

On the day of the challenge, both viruses were thawed and diluted in cell culture medium (DMEM, Dulbecco’s Modified Eagle’s Medium) to match the target titer of 10^4^ TCID_50_/2 mL. This target titer corresponded to the target titer used in several Zoetis laboratory efficacy challenge studies conducted in the past using the Olot/91 strain and induced severe reproductive disease in PRRSV-naïve sows. The virus was kept on ice during the challenge process. Pre- and post-challenge titrations were performed in PAM and MA-104. 

### 2.3. Animals and Experimental Design

Fifteen clinically healthy, cross-bred pregnant female pigs without a history of disease caused by PRRSV or vaccination against the same organisms were enrolled at 85–95 days of gestation and assigned to treatments. 

On Day 0, sows were intranasally (IN) inoculated with 2 mL (1 mL in each nostril) of the corresponding treatment: NTX (*n* = 4) not infected; T01 (*n* = 5) infected with PRRSV Olot/91 strain; T02 (*n* = 6) infected with Horsens strain. The animals were monitored during the remaining gestation and allowed to farrow naturally.

Treatments, NTX and T01, were allocated to sows according to a random treatment allocation plan and randomly assigned to challenge rooms and crates. Sows belonging to T02 were not randomly selected or allocated to the room but were randomized to crates within the room. Sows with later pregnancy dates were assigned to the T02 group in order to allow more time to obtain the Horsens strain (the study was started while the recombinant virus strain was not yet available at the study site).

Each treatment group was housed in an independent facility to avoid cross-contamination. NTX animals were kept in the farm of origin as a negative control reference for reproductive performance.

Sows were fed once a day with pelleted feed. Water was provided ad libitum. Piglets were housed with the sow from birth to the end of the study. The general health of the animals was observed and recorded daily during the whole study. Blood and nasal swab samples were collected from T01 and T02 sows every 2–3 days during 10 days after challenge in order to analyze viremia and nasal shedding by PRRSV RT-qPCR. The incidence of abortions during the post-challenge period and reproductive performance at farrowing was evaluated. 

A blood sample was taken from T01 and T02 alive piglets at birth and at weaning to evaluate viremia (vertical transmission). Viral load was evaluated (PRRSV RT-qPCR) in lung exudates at birth from stillborns. Pre-weaning mortality, lung macroscopic lesions at weaning, and viral load in lung bronchoalveolar lavage (BAL) at weaning were also evaluated.

### 2.4. Reproductive Data

For each sow, the following reproductive variables were recorded: date of abortion/farrowing and litter details. On the litter details, the following assessments were recorded: the number of piglets born alive (healthy plus low-viability piglets); healthy piglets; low-viability piglets; stillborn piglets; mummies (autolytic partly or completely mummified fetus). Piglets born alive were identified with ear tags. At 21 ± 2 days post-farrowing, the number of weaned piglets (live piglets per litter) was recorded. All piglets that died or were euthanized for welfare reasons during the study were also recorded.

### 2.5. Sampling

Blood samples and nasal swabs were taken from all sows before challenge (Day 0) and then every 2–3 days until Day 10 of the study. Blood samples were taken from alive piglets (T01 and T02 groups) at farrowing and at weaning (21 ± 2 days post-farrowing). Blood samples were kept for at least two hours at room temperature and then centrifuged for ten minutes at 2500× *g* ± 100 g at room temperature. The serum was stored at −80 ± 10 °C until tested. The nasal swab tips were placed into Eppendorf tubes with 1 mL of sterile PBS (phosphate-buffered saline) and stored at –80 ± 10 °C until tested.

Lung exudates of aborted or stillborn piglets (T01 and T02 groups) were obtained at farrowing by keeping the lungs O/N at 5 ± 3 °C in a sterile container. The following day, the exudates were removed and stored at −80 ± 10 °C until further analysis.

Bronchoalveolar lavages were collected from all piglets that were euthanized on Day 21 ± 2 days post- farrowing (T01 and T02 groups). At necropsy, after lung lesions evaluation, 10 mL of PBS was introduced through the trachea inside the lungs; after a mild lung massage, PBS was recovered into sterile tubes by decantation and stored at −80 ± 10 °C until further analysis.

### 2.6. Euthanasia and Lung Scoring

Piglets from T01 and T02 groups were euthanized on Day 21 ± 2 days post-farrowing (at weaning) with an overdose of sodium pentobarbital (≥100 mg/kg IV), followed by exsanguination. NTX sows and piglets were returned to the farm stock.

The lungs were removed from the piglet carcass, and the lung lesions were scored as the percentage of consolidation for each lobe (left cranial, left middle, left caudal, right cranial, right middle, right caudal, and accessory).

### 2.7. Titration

Olot/91 strain was tittered by endpoint dilution on swine pulmonary alveolar macrophages (PAM) plated at 3 × 10^5^ cells/mL in 96-well plates with DMEM supplemented with 5% FetalClone Serum III (GE Healthcare, Chicago, IL, USA). Fifty microliters of ten-fold dilutions of the titration sample were dispensed per well, with 8 replicates per dilution. DMEM with 5% FetalClone III and 0.1% PEG (penicillin, streptomycin, and gentamycin) was used as a negative control. The plate was incubated at 37 °C and 5% of CO_2_ for 7 days. Then, the presence or absence of cytopathic effect was determined using an optical microscope (Olympus Europa SE & Co, Hamburg, Germany). The viral titer was determined following Spearman and Kärber formula [18]. 

Horsens strain was tittered by end-point dilution on MA-104 cells plated at 3 × 10^5^ cells/mL in 96-well plates with MEM-E supplemented with 5% of FetalClone Serum III (GE Healthcare). Fifty microliters/well of ten-fold dilution of the titration sample were dispensed per well, with 8 replicates per dilution, and incubated at 37 °C and 5% CO_2_ for 7 days. Then, the presence or absence of cytopathic effect was determined using an optical microscope (Olympus Europa SE & Co). The viral titer was determined following Spearman and Kärber formula [18].

### 2.8. Serology

Sow sera collected pre-challenge (Day 0) was tested for antibodies to PRRSV using the IDEXX PRRS X3 Ab Test (IDEXX, Westbrook, ME, USA), following manufacturer’s instructions.

### 2.9. Real-Time Quantitative Polymerase Chain Reaction (RT-qPCR)

Viremia (PRRSV load in serum), shedding (PRRSV load in nasal swabs), and viral load in BAL lavages and lung exudate samples were measured by RT-qPCR. 

Total RNA was extracted from the samples using the Biosprint 96 DNA blood kit. The purified viral RNA was reverse transcribed at 50 °C for 30 min and denatured at 95 °C for 5 min. The PCR program of reactions consisted of 40 cycles of denaturation at 95 °C for 20 s and annealing at 53 °C for 40 s. The RT-qPCR was conducted in a 7500 Real-Time PCR System thermal cycler.

The oligonucleotide primers and dual-labeled probe used for amplification were 5’-GCACCACCTCACCCAGAC-3’ (forward, final concentration 0.5 μM); 5’ CAGTTCCTGCGCCTTGAT-3’ (reverse, final concentration 0.5 μM); 4’-6-FAM-CCTCTGCTTGCAATCGATCCAGAC-BHQ_1_-3’ (dual-labeled probe, final concentration 0.6 μM), which correspond to base-pair positions 14,792-14,809, 14,851-14,868, and 14,819-14,842, respectively, of the EU prototype strain Lelystad (Genbank accession number M96262). The amplicon consists of a 77-bp fragment from ORF7.

The genome equivalents (RNA copy number per 5 μL) were interpolated from the RNA standard curve for this assay and adjusted (RNA copy number per 1 mL of the sample) according to the sample dilution.

### 2.10. Statistical Analysis

Data summaries and analyses were performed with a centralized data management system (SAS/STAT User’s Guide V. 9.4, SAS Institute, Cary, NC, USA). The room was the experimental unit for all the statistical analyses, with animals as subsamples. There was only one experimental unit per treatment. Thus, no statistical analyses were performed. The percent of piglets from each litter, which were live births, healthy (live piglets minus low-viability), low-viability, stillborn, mummies, and pigs weaned at Day 21 ± 2 after birth (number of pigs weaned/number of pigs born alive), was calculated. The percent of the litter that was normal (healthy), stillborn, mummies, low-viability, and weaned was transformed with an arc sin square root transformation and summarized with descriptive statistics, back-transformed means, standard deviations, and ranges for each treatment. Prior to the summary, the RT-qPCR data (sow viremia, sow shedding, piglet viremia) was transformed using an appropriate logarithm transformation. Transformed viremia data (sow and piglet) was summarized with descriptive statistics, back-transformed means, standard deviations, and ranges for T01 and T02 treatments. Sow serology data was transformed and summarized with descriptive statistics. Furthermore, frequency distributions of positive/negative results were calculated for each treatment (NTX, T01, and T02). Negative samples were given a value of 50 PRRSV RNA copies/mL (1.7 log_10_ PRRSV RNA copies/mL), which corresponds to a half of the quantification limit of the technique (100 PRRSV RNA copies/mL).

Finally, the percentage of total lung with lesions was calculated using the following formula: Percentage of total lung lesions = (0.10 × left cranial) × (0.10 left middle) + (0.25 × left caudal) + (0.10 × right cranial) + (0.10 × right middle) + (0.25 × right caudal) + (0.10 × accessory). The arcsine square root transformation was applied to the percentage of the total lung with lesions prior to the summary. The transformed lung lesions were summarized with descriptive statistics, back-transformed means, standard deviations, and ranges for each treatment (T01, T02).

## 3. Results

### 3.1. Reproductive Performance of Sows

Table 1 summarizes the reproductive performance of sows by treatment. No animal aborted throughout the study. NTX sows farrowed 89.8% of alive piglets, whereas 36.0% of piglets born from T01 (Olot/91 strain) sows and 49.9% of piglets born from T02 (Horsens strain) sows were born alive. Seventy-eight percent of NTX, 27.1% of T01, and 41.7% of T02 were healthy piglets, whereas 8.8% of NTX, 0.9% of T01, and 2.9% of T02 were low-viability piglets. At farrowing, NTX sows had 10.3% of stillborn piglets compared to 64% of T01 and 50.1% of T02 groups. No mummies were recorded in any treatment group.

89.9% of animals born form NTX sows were weaned compared to 49% of piglets born from T01 sows and 53.2% of piglets born from T02 sows.

### 3.2. Sows Viremia

All sows were confirmed as PRRSV-naïve (serum-negative for the presence of antibodies by ELISA) at Day 0. T01 animals (infected with Olot/91 strain) were all positive for PRRSV by RT-qPCR at Day 3 post-infection and remained viremic until Day 10 (last sampling day) (Table 2). One animal (16.7%) from T02 was positive at Day 3, two at Day 6 (33.3%), and three at Day 10 (50.0%). Overall, 50.0% of T02 sows remained free of viremia at Day 10 post-infection.

### 3.3. Sows Nasal Shedding

All sows from the T02 group were found RT-qPCR PRRSV-negative in nasal swabs at Day 0 (day of the challenge) (Table 3). Four out of five sows from the T01 group were found negative in nasal swabs prior to the challenge. One sow was found positive, which is most likely related to cross-contamination during laboratory analysis as it was surrounded by samples with a high viral load on the PCR plate. Furthermore, the result obtained on the following sampling day (Day 3) was found negative in this sow.

All animals infected with T01 (Olot/91 strain) presented nasal shedding post-infection, whereas only 50% of T02 sows were positive at some point during the 10-day post-challenge observation. 

### 3.4. Piglet Viremia

At birth (within 24 h post-farrowing), 98.91% of piglets born from T01 sows were viremic, while only 64.99% of piglets from T02 were positive in serum at that time (Table 4). At weaning (21 ± 2 days post-farrowing), the prevalence of positive piglets was 100% and 50.0% in the T01 and T02 groups, respectively.

The geometric mean amount of viral load detected in blood at farrowing was 1.83 × 10^6^ copies/mL in T01 piglets, whereas the geometric mean viral load in blood in T02 piglets was 1.64 × 10^3^ copies/mL. The geometric mean viral load in blood at weaning increased to 1.05 × 10^7^ copies/mL for T01 piglets and 1.95 × 10^3^ copies/mL for T02 piglets.

### 3.5. Viral Load in Bronchoalveolar Lavages (BAL)

All pigs weaned from T01 sows (16/16) were PRRSV-positive in BAL compared to only 28.57% (6/21) of pigs weaned from T02 sows (Table 5).

The geometric mean amount of virus detected in BAL samples was 1.11 × 10^6^ copies/mL in the T01 group (Olot/91) and 8.39 × 10^2^ copies/mL in the T02 group (Horsens).

### 3.6. Viral Load in Lung Exudates (LE)

Thirty-eight of 45 (84.44%) stillborn piglets from T01 sows were PRRSV-positive, whereas only 29 of 43 (67.44%) stillborn piglets born from T02 sows were positive (Table 6). The geometric mean amount of virus detected in LE samples was 5.30 × 10^6^ copies/mL in T01 and 1.71 × 10^5^ copies/mL in stillborn T02 piglets.

### 3.7. Lung Lesions in Piglets

At necropsy (21 ± 2 days post-farrowing), 11/16 piglets (68.8%) born from T01 sows had macroscopic lung lesions consistent with PRRSV-1 infection. In contrast, only 8/21 (38.1%) piglets from the T02 group had macroscopic lung lesions. In both treatments, the lesions observed were considered mild, with a back-transformed mean of 0.9% for T01 and 0.1% for T02 (Table 7) of affected lung parenchyma.

## 4. Discussion

The Horsens strain was isolated from serum samples from infected sows that received PRRSV contaminated semen from a boar station. That boar station was PRRSV seronegative until July 2019, when PRRSV-1 was detected in samples taken during routine PRRSV surveillance [16]. It was hypothesized that the source of infection was a neighboring farm situated 5.8 km from the boar station and that the PRRSV-1 virus isolated (Horsens strain) resulted from homologous recombination between two PRRSV-1 MLV vaccine strains [16]. The major parental vaccine strain is Suvaxyn PRRS MLV (Zoetis, Louvain-la-Neuve, Belgium), and the minor parental vaccine strain is Unistrain PRRS (Hipra, Amer, Spain); however, the high-resolution analysis revealed that a small segment of the Horsens virus might originate from a field strain.

The safety and effectiveness of a vaccine in the field depend not only on the properties of the vaccine itself but also on how it is applied and what other biosecurity measures are in place [19]. MLVs should be used following guidelines in order to maintain their safety: they should not be used in PRRS-naïve herds or in pigs that are not healthy, and only pigs of the recommended age and stage of pregnancy should be vaccinated. If two or more PRRSV strains are contemporaneously present in the same herd, a possibility of recombination exists [20,21]. Recombination between PRRSV strains has been described under laboratory and field conditions [22,23]. Recombination events are not exclusive to PRRSV field strains; these events have also been described involving vaccine strains [10,13,14,15].

In the present study, the impact of a PRRSV recombinant strain (Horsens) on the reproductive performance of seronegative (naïve) pregnant sows in the last third of gestation (85–95 days of gestation) was evaluated.

The challenge was assessed based on sow and piglet data. Sow data included reproductive performance and virological data. Piglet data included virological and pathological data.

Viremia after challenge should provide the confirmation that the sows had been properly infected. In the present study, all sows inoculated with the strain Olot/91 developed viremia within a 10-day period, while only 50% of the sows inoculated with the Horsens strain did so. Similar results were observed for nasal shedding. Due to the setup of the experiment, the results could not be statistically compared; however, the amount of virus detected was numerically higher (at least 256-fold higher at day 10 in the blood) in Olot/91 than Horsens inoculated sows. 

Although 50% of T02 sows did not have any detectable viral load in serum or nasal swabs during the 10-day observation period after challenge, they all farrowed piglets with confirmed positive viremia. One explanation could be that these sows had a very short viremia that was not picked up in the serial bleedings (for instance, if it occurred at days 8 and 9 after challenge). This is considered unlikely as serum viremia may last for several weeks [24]. The other explanation is that Horsens inoculated sows had a delayed onset of replication (compared to Olot/91 inoculated sows) [25] and/or became positive later due to shedding from pen mates [19]. This latter hypothesis is considered more likely. For example, one of the Horsens inoculated sows was viremic only on Day 10.

PRRSV-associated reproductive disorders are characterized by an increased rate of premature farrowing, late-term abortions, stillborn or weak piglets, and mummified fetuses [26,27,28]. In the present study, neither abortions nor mummies were observed. Two out of six sows infected with Horsens strain farrowed prematurely, resulting in stillborn and low-viability animals. From those sows, only one piglet was weaned. Both Olot/91 and Horsens inoculated sows farrowed fewer live piglets than NTX sows (36%, 49.9%, and 89.7%, respectively). From all farrowed piglets, only 49% of the pigs from sows challenged with Olot/91 and 53.3% of Horsens were weaned. The NTX group weaned 89.9% of piglets. Thus, the Horsens virus behaved similarly, from a reproductive point of view, to the field strain (Olot/91). Reports of exacerbated virulence reported by some Danish farmers [16] were not confirmed under these experimental conditions. In contrast, the Horsens group had more pigs born alive (49.9% vs. 36.0%) and more pigs weaned (53.2% vs. 49.0%) than the Olot/91 group. 

The virologic data collected from the Olot/91 and Horsens offspring further supported the reduced virulence of the Horsens strain compared to the field strain Olot/91. Due to the experimental design (the experimental unit was the room, not the animal), it was not possible to analyze the data using an ANOVA. However, piglets infected with the recombinant strain showed a viremia level of 1000- to 5000-fold lower in comparison to the Olot/91-infected piglets in blood samples; 1000-fold lower in BALs and 31-fold lower in lung exudates. These results did not confirm that serum from Horsens-infected pigs exceeded viral loads normally seen in samples from PRRSV-diseased pigs [16].

Furthermore, the back-transformed mean percentage of the affected lung of Olot/91 piglets was 0.9%, whereas the affected lung of Horsens piglets was 0.1%. Overall, 68.8% of Olot/91 piglets and 38.1% of Horsens piglets presented mild lung lesions. Lungs presented a multifocal greyish patchy pattern, which was consistent with subacute interstitial pneumonia, the typical lesion induced by PRRSV-1 in young pigs, although it was not confirmed by histopathology. These results confirmed the similarity of Horsens strain to other PRRSV-1 subtype 1 strains in inducing mild respiratory pathology in infected pigs [29,30,31] and did not confirm reports of a significant increase in respiratory disease reported in some Danish herds [16].

The sequencing of the Horsens strain has previously shown that the parental strains are the Suvaxyn PRRS MLV (Zoetis, Louvain-la-Neuve, Belgium) and Unistrain PRRS (Hipra, Amer, Spain) strains [16]. The results from the present study indicated similar pathogenicity of the Horsens strain compared to a well-characterized Lelystad-like field strain. Although MLVs are currently the most effective option in the market to control PRRS [32], they have the intrinsic risk of recombination under farm conditions [33]. To limit the occurrence of recombination between vaccine strains, the use of multiple live PRRS vaccines in a pig flow, either simultaneously and or in rapid succession, should be avoided. When changing from one MLV to another, it is important to allow the first vaccine time to reduce its titer and prevalence before introducing the second vaccine. Care should be taken to eliminate or minimize opportunities for pigs to become infected by both vaccine viruses.

Finally, the epidemiological, virological, and clinical data from Denmark indicated that Horsens strain had regained a profound level of virulence despite the fact that the two parent viruses were attenuated vaccine strains. However, the current study could not confirm that this strain was particularly virulent from a clinical and virological point of view. This study was not designed to address the reproductive virulence of the recombinant virus relative to the parental vaccine strains. It would come as no surprise if the Horsens strain caused greater reproductive disease than either parental vaccine strain would if used off-label in PRRS-naïve sows late in pregnancy. The recombination process allows key attenuating mutations in one vaccine virus to be replaced with the corresponding non-mutated sequence from the other. However, all licensed PRRS MLVs are contraindicated for use in sow herds containing PRRS-negative pregnant sows because they induce residual reproductive virulence. Demonstrating statistically significant differences between the vaccines and recombinant would require a dedicated study.

## 5. Conclusions

The recombinant PRRSV strain Horsens yielded reproductive performance results that were numerically less compared to those reported for a typical PRRSV-1 subtype 1 strain when administered to PRRSV seronegative sows in the last third of gestation. In the present study, the Horsens strain was apparently less virulent than the field strain Olot/91 under laboratory conditions.

## Figures and Tables

**Table 1:** Back-transformed mean (±SD) and range of different reproductive parameters by treatment.

	Treatment					
	NTX		T01		T02	
	Mean ± SD	Range	Mean ± SD	Range	Mean ± SD	Range
At farrowing, %						
Abortion	0		0		0	
Born alive	89.7 ± 14.03	73.91 to 100	36.0 ± 13.50	21.05 to 55.56	49.9 ± 18.55	27.27 to 71.43
Born healthy	78.0 ± 12.20	69.57 to 98.28	27.1 ± 28.81	0 to 55.56	41.7 ± 18.39	27.27 to 71.43
Low-viability	8.8 ± 5.37	4.35 to 17.65	0.9 ± 4.04	0 to 21.05	2.9 ± 8.97	0 to 28.57
Stillborn	10.3 ± 14.03	0 to 26.09	64.0 ± 13.50	44.44 to 78.95	50.1 ± 18.55	28.57 to 72.73
Mummies	0		0		0	
Weaned, %	89.8 ± 14.16	73.33 to 100	49.0 ± 47.97	0 to 80	53.2 ± 72.28	0 to 100

NTX: not infcted; T01: Olot/91; T02: Horsens strain. SD: standard error.

**Table 2:** Geometric mean, SD, and range of viral load in sow serum after a challenge by treatment and time-point.

Treatment	Day of Study	Geometric Mean (Copies/mL)	SD (Copies/mL)	Range (Copies/mL)
T01	D3	1.20 × 10^6^	9.37 × 10^5^	3.90 × 10^5^ to 2.82 × 10^6^
	D5	4.06 × 10^6^	7.33 × 10^6^	2.92 × 10^5^ to 3.85 × 10^7^
	D7	1.73 × 10^5^	3.38 × 10^5^	4.13 × 10^4^ to 5.07 × 10^6^
	D10	5.54 × 10^6^	5.60 × 10^6^	2.07 × 10^6^ to 2.00 × 10^7^
T02	D3	2.82 × 10^2^	1.19 × 10^3^	50 to 1.61 × 10^6^
	D6	2.27 × 10^3^	1.36 × 10^4^	50 to 1.95 × 10^7^
	D10	2.16x 10^4^	1.46 × 10^5^	50 to 8.97 × 10^7^

T01: Olot/91; T02: Horsens strain; SD: standard deviation. Values are expressed as PRRSV RNA copies per mL of serum. D, day of study. Viremic if >50 PRRSV RNA copies/mL.

**Table 3:** Geometric mean, SD, and range of sow nasal shedding pre- and after a challenge by treatment and time-point.

Treatment	Day of Study	Mean (Copies/mL)	SD (Copies/mL)	Range (Copies/mL)
T01	D0	87.0	1.07 × 10^2^	50 to 7.96 × 10^2^
	D3	6.72 × 10^3^	3.18 × 10^4^	50 to 9.15 × 10^5^
	D5	6.40 × 10^4^	8.20 × 10^4^	1.81 × 10^4^ to 3.69 × 10^5^
	D7	2.81 × 10^4^	1.34 × 10^4^	1.29 × 10^4^ to 4.89 × 10^4^
	D10	3.90 × 10^2^	1.16 × 10^3^	50 to 3.31 × 10^4^
T02	D0	50.0	0	50 to 50
	D3	98.9	1.05 × 10^2^	50 to 4.91 × 10^2^
	D6	3.41 × 10^2^	1.11 × 10^3^	50 to 1.31 × 10^5^
	D10	2.11 × 10^3^	8.97 × 10^3^	50 to 3.84 × 10^5^

T01: Olot/91; T02: Horsens strain; SD: standard deviation. Values are expressed as PRRSV RNA copies per mL. D, day of study. Viremic if > 50 PRRSV RNA copies/mL.

**Table 4:** Geometric mean (±SD) of viral load in serum at farrowing and weaning and back-transformed percentage of viremic piglets by treatment.

Treatment	Day of Study			
	Farrowing		Weaning	
	Viral Load	% Viremic Pigs	Viral Load	% Viremic Pigs
T01	1.83 × 10^6^ ± 9.05 × 10^6^	98.91%	1.05 × 10^7^ ± 2.21 × 10^7^	100.00%
T02	1.64 × 10^3^ ± 7.62 × 10^3^	64.99%	1.95 × 10^3^ ± 1.17 × 10^4^	50.00%

T01: Olot/91; T02: Horsens strain; SD: standard deviation. Values are expressed as PRRSV RNA copies per mL. D, day of study. Viremic if >50 PRRSV RNA copies/mL.

**Table 5:** Geometric mean (±SD) of viral load in BAL and the percentage of RT-qPCR positive piglets by treatment.

Treatment	Viral Load (Mean ± SD)	% Viremic Pigs
T01	1.11 × 10^6^ ± 2.38 × 10^6^	100%
T02	8.39 × 10^2^ ± 3.92 × 10^3^	28.57%

T01: Olot/91; T02: Horsens strain; SD: standard deviation. Values are expressed as PRRSV RNA copies per mL. D, day of study. Viremic if >50 PRRSV RNA copies/mL.

**Table 6:** Geometric mean (±SD) of viral load in lung exudates and the percentage of RT-qPCR positive piglets by treatment.

Treatment	Viral Load (Mean ± SD)	% Viremic Pigs
T01	5.30 × 10^6^ ± 2.82 × 10^7^	84.44%
T02	1.71 × 10^5^ ± 1.09 × 10^6^	67.44%

T01: Olot/91; T02: Horsens strain; SD: standard deviation. Values are expressed as PRRSV RNA copies per mL. D, day of study. Viremic if >50 PRRSV RNA copies/mL.

**Table 7:** Back-transformed mean (± SD) percentage of lung lesions and piglets with lesions by treatment.

Treatment	Mean % Lung Lesions	% Of Animals with Lesions
T01	0.9 ± 2.47	68.8%
T02	0.1 ± 0.89	38.1%

T01: Olot/91; T02: Horsens strain; SD: standard deviation.
